# A high concentration CO_2_ pool over the Indo-Pacific Warm Pool

**DOI:** 10.1038/s41598-023-31468-0

**Published:** 2023-03-15

**Authors:** R. Peter, J. Kuttippurath, Kunal Chakraborty, N. Sunanda

**Affiliations:** 1 CORAL, Indian Institute of Technology Kharagpur, Kharagpur, West Bengal 721302 India; 2 Indian National Centre for Ocean Information Services, Ministry of Earth Sciences, Hyderabad, India

**Keywords:** Carbon cycle, Atmospheric chemistry

## Abstract

Anthropogenic emissions have produced significant amount of carbon dioxide (CO_2_) in the atmosphere since the beginning of the industrial revolution. High levels of atmospheric CO_2_ increases global temperature as CO_2_ absorbs outgoing longwave radiation and re-emits. Though a well-mixed greenhouse gas, CO_2_ concentration is not uniform in the atmosphere across different altitudes and latitudes. Here, we uncover a region of high CO_2_ concentration (i.e. CO_2_ pool) in the middle troposphere (500–300 hPa) over the Indo-Pacific Warm Pool (IPWP, 40° E–140° W, 25° S–25° N), in which the CO_2_ concentration is higher than that of other regions in the same latitude band (20° N–20° S), by using CO_2_ satellite measurements for the period 2002–2017. This CO_2_ pool extends from the western Pacific to the eastern Indian Ocean. Much of the CO_2_ pool is over the western Pacific Ocean (74.87%), and the remaining lies over the eastern Indian Ocean (25.13%). The rising branch of Walker circulation acts as a “CO_2_ Chimney” that constantly transports CO_2_ released from the natural, human-induced and ocean outgassing processes to the middle and upper troposphere. The CO_2_ pool evolves throughout the year with an average annual trend of about 2.17 ppm yr^−1^, as estimated for the period 2003–2016. Our analysis further reveals that La Niña (El Niño) events strengthen (weaken) the CO_2_ pool in the mid-troposphere. The radiative forcing for the CO_2_ pool suggests more warming in the region and is a grave concern for global warming and climate change.

## Introduction

Carbon dioxide (CO_2_), a major greenhouse gas (GHG), concentration has been steadily rising in the atmosphere since the mid-nineteenth century. The global warming due to high levels of GHGs might increase surface temperatures over 1.5 °C above the pre-industrial levels by 2030^1,2^. This accelerated warming is worrisome, as it leads to more frequent and severe extreme events such as heatwaves^3,4^, floods^5,6^, and changes in tropical cyclone activity^7,8^ and rainfall patterns^9,10^ with devastating economic and environmental consequences^11^. The atmospheric CO_2_ levels climbed up to 412 ppm in January 2020, with an average global trend of 2.11 ppm yr^−1^ during the period 2003–2016^12–14^.

A thorough understanding of the dynamics, evolution, fate and human influence on atmospheric CO_2_ is essential to enact effective carbon mitigation policies. The spatial and temporal variations of atmospheric CO_2_ show distinct annual, seasonal and latitudinal gradients^15^. There are substantial differences in CO_2_ concentrations between continents and oceans, which are dependent on the sources of emissions. Large-scale atmospheric circulations, weather systems and jet streams distribute CO_2_ around the globe^15^. Northern Hemisphere (NH) is known for higher levels of atmospheric CO_2_ than that of the Southern Hemisphere (SH). In NH, seasonal changes in CO_2_ are primarily driven by the result of metabolic activity of terrestrial plants and soils^16^. The warmer climate alters the seasonal CO_2_ cycle^17^. The effect of rising atmospheric CO_2_ increases radiative forcing, which leads to higher sea surface temperature (SST)^18^. These also cause a reduction in surface-to-deep ocean transport of CO_2_ and a reduction in oceanic carbon, which might increase the concentration of atmospheric CO_2_^19^.

An oceanic region enclosed by 28 °C or higher SST isotherm is known as a warm pool^20^. These are the regions with high precipitation, strong atmospheric convection and surface wind convergence^21^. The warm pool regions of western tropical Pacific Ocean and the tropical Indian Ocean constitute the Indo-Pacific Warm Pool (IPWP)^22^. Previous studies suggest that warmer SST in IPWP strengthens the upwelling branch of Hadley circulation and weakens (strengthens) its downwelling branch in NH (SH). Furthermore, due to the rapid warming of Indian Ocean, the westward extension of IPWP shifts the walker circulation westward, which decreases subsidence over eastern Africa and makes the region drier^23,24^.

IPWP has a significant role in transporting surface emissions to higher altitudes over the tropics. Such a system, which is a source of heat and moisture, prevails throughout the year regardless of seasons in West Pacific and East Indian Ocean (EIO) in the tropics. IPWP shows meridional and zonal variability that can influence global atmospheric circulation, and onset, intensity and duration of climate modes such as El Niño Southern Oscillation (ENSO)^25^. As compared to the oscillations of the eastern edge of western Pacific Warm Pool (WPWP), it is observed that South and North Warm Tongues are dominated by the annual SST cycles and are influenced by the El Niño onset, but East Cold SST Tongue is totally dominated by the El Niño onset and shows no distinct annual cycle^26^. Yan et al.^27^ studied the temperature and size variations of WPWP, and found that changes in solar irradiance, ENSO events and global warming could have modified the distribution of SST and the size of IPWP. The warm pool in Indian Ocean has a stronger annual cycle than that of Pacific^28^. Furthermore, SST in Indian Ocean is rising at a faster rate than in other tropical ocean regions^29,30^.

During El Niño, warmer than normal subsurface water in the tropical Pacific replaces the cooler CO_2_-rich water^31^. It reduces or reverses the CO_2_ released by tropical oceans and increases the uptake of global-oceanic CO_2_^32^. For instance, surface measurements show a reduction in CO_2_ outgassing during the 2015–2016 El Niño event^33^. Therefore, oceans act as CO_2_ sink during El Niño and thus, they reduce CO_2_ concentrations in the atmosphere when the tropical SST is warmer^17^.

This study is organized as follows: we analyze the anomalously high values of mid-tropospheric CO_2_ over IPWP using satellite data. We examine possible reasons for the high CO_2_ region and its radiative forcing. We also investigate the influence of ENSO on the CO_2_ concentrations in the mid-troposphere. We supplement our analysis using buoy measurements to assess changes in near-surface CO_2_, which has a critical role in contributing to the high CO_2_ region.

## Results

### Global distribution of atmospheric CO_2_

**Figure 1:**
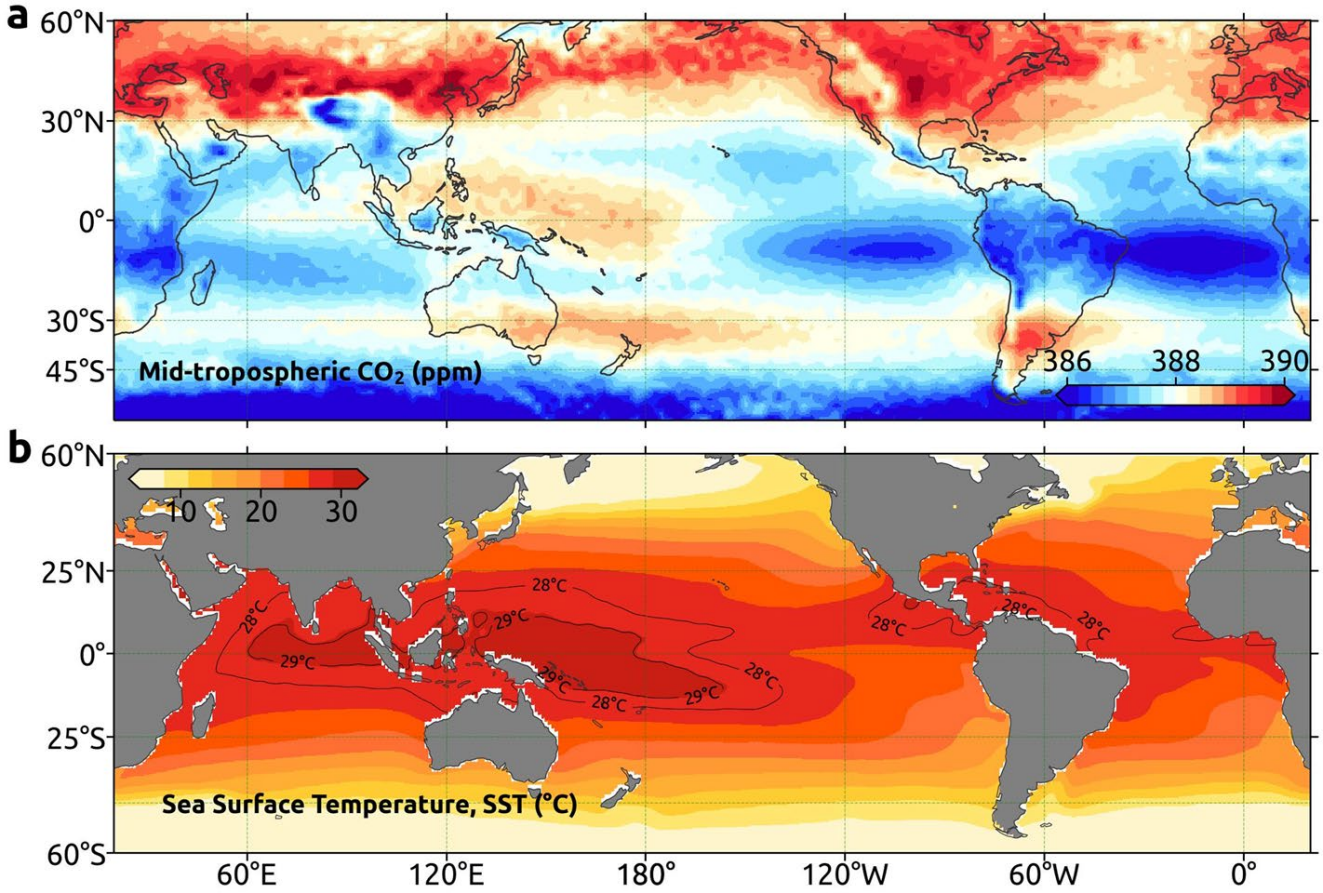
Atmospheric CO_2_ and Sea Surface Temperature. Annual averaged (**a**) mid-tropospheric CO_2_ (**b**) HadISST Sea Surface Temperature (SST) for the period 2002–2017. SST contours of 28 °C and 29 °C are overlaid. These maps are generated using Cartopy^64^ 0.18.0 (https://scitools.org.uk/cartopy).

Figure 1a shows a region of high CO_2_ concentration between 30° N and 60° N and comparatively lower concentration in the rest of the region. The largest seasonal difference is observed in the 45°–60° N region (3.21 ppm) and lowest in 0°–30° N (1.32 ppm), as shown in Supplementary Figure S1. North America and Europe exhibit the highest CO_2_ concentration in 45°–60° N, whereas the largest CO_2_ concentration over Asia is found in 30°–45° N. The highest difference is observed between the regions 30°–60° N and 0°–30° N, with its peak in 2010 (1.81 ppm, Supplementary Figure. S1). There is an increase of 5.8% in global CO_2_ emissions, with a peak of 33 billion tonnes in 2010 due to the continued growth of developing countries and economic recovery in developed nations^34^, which is also the reason for the highest difference in 2010.

The average of CO_2_ over land regions during 2003–2016 is 388.52 ± 0.93 ppm, whereas it is 387.95 ± 1.27 ppm over the oceans. Also, there is a difference of 1.13 ± 0.1 ppm between land regions of the north and south hemispheres, and 0.63 ± 0.06 ppm between the oceans of both hemispheres. Higher emission regions show higher CO_2_ concentrations (e.g. NH), but lower over the carbon sink regions (e.g. the Atlantic minimum). Note that atmospheric circulation also plays a key role in the redistribution and mixing of CO_2_^15^. As illustrated in Fig. 1a, the southern Atlantic and Pacific Oceans have the low CO_2_ zones among the regions, which can be attributed to the presence of large carbon sinks, atmospheric subsidence and absence of CO_2_ sources there^12^. The anomaly between the global ocean CO_2_ and low CO_2_ regions of the Atlantic and Pacific Oceans shows differences larger than 1 ppm, although it decreases after 2010 (Supplementary Figure. S1). The decreasing tendency in CO_2_ anomalies at these low CO_2_ regions indicates that the minimum zones are shrinking and it could be the repercussions of deforestation in the Amazon rainforest and climate change^35^.

The regional temperature exhibits a positive feedback on the atmospheric concentrations of CO_2_. A major region of heat source in the ocean, the IPWP, acts as a medium to distribute CO_2_ in the atmosphere to different altitudes^12^. The global distribution of SST is shown in Fig. 1b.

### A high CO_2_ pool over the tropical ocean

**Figure 2:**
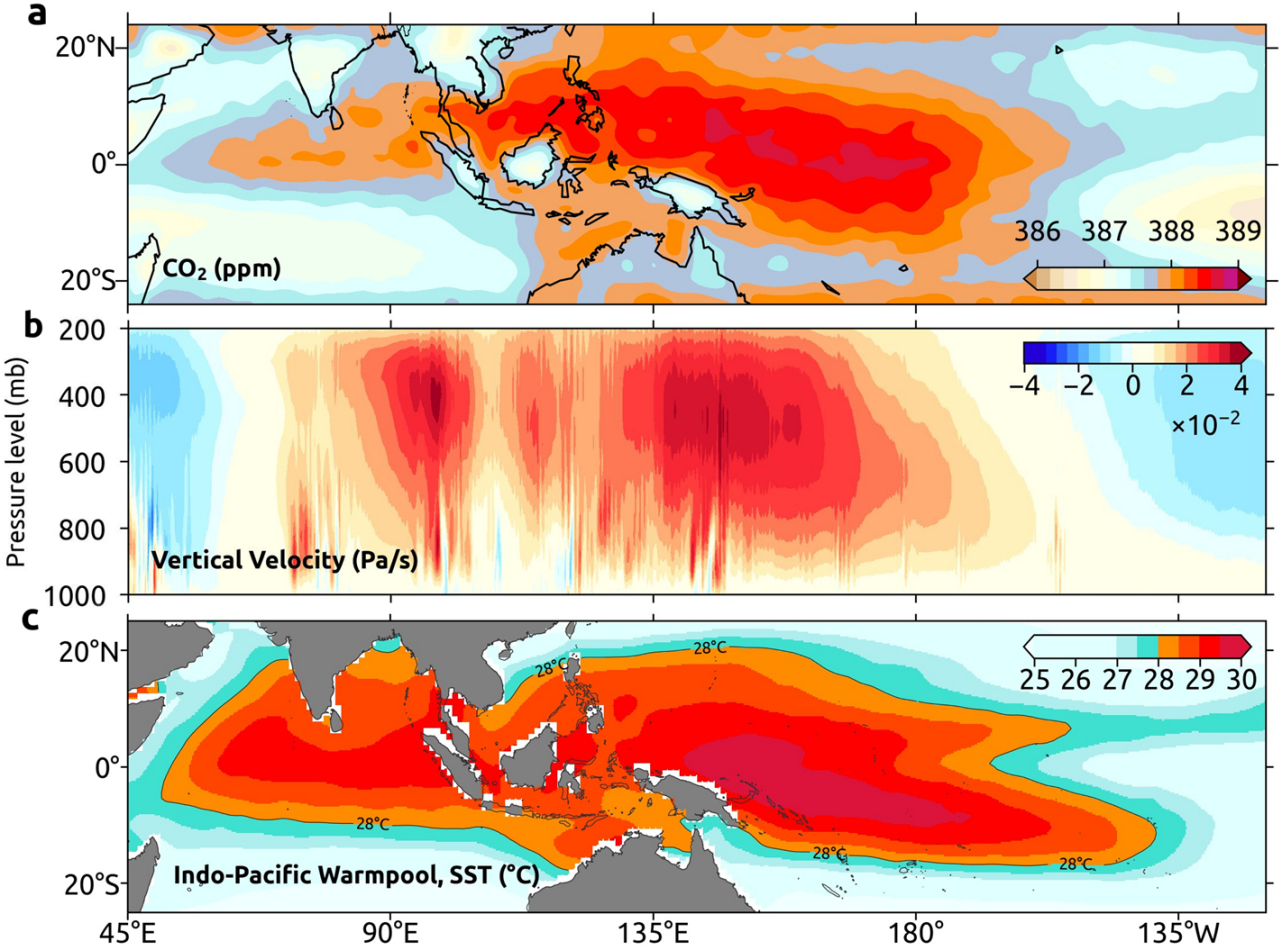
CO_2_ pool and its driving factors. (**a**) Annual-averaged (2002–2017) mid-tropospheric CO_2_ with the CO_2_ pool region. (**b**) Annual and latitude (20° S – 20° N) averaged vertical velocity for the period 2002–2017 and (**c**) Annual-averaged (2002–2017) Sea Surface Temperature (SST) in the Indo-Pacific region. Here SST contour of 28° C represents the Indo-Pacific Warm Pool (IPWP). These maps are generated using Cartopy^64^ 0.18.0 (https://scitools.org.uk/cartopy).

It is interesting to note a zone of high CO_2_ concentration in the mid-troposphere over IPWP, although CO_2_ concentrations over tropical regions are relatively lower than that in the mid-latitudes of NH^36^. Hereafter, it is referred to as “the CO_2_ pool”, which is shown in Fig. 2a. The CO_2_ pool stretches from the tropical East Indian Ocean to the tropical central Pacific Ocean, with a major part over the latter region.

Figure 2b shows the region-averaged (20° S–20° N) vertical velocity for the period 2002–2017. The positive values between 60° E and 150° W indicate the ascending arm of Walker circulation and the negative values between 120° W and 90° W show its descending branch in Pacific Ocean. The upwelling air over IPWP (Fig. 2c) brings high CO_2_ from the surface to mid-troposphere, which makes high CO_2_ concentrations there. However, the sinking air over the eastern Pacific Ocean brings low CO_2_ to the mid-troposphere from higher altitudes. The descending arm of Walker circulation over the western Indian Ocean brings lower CO_2_ concentration in these regions.

### Variability of Indo-Pacific CO_2_ pool

**Figure 3:**
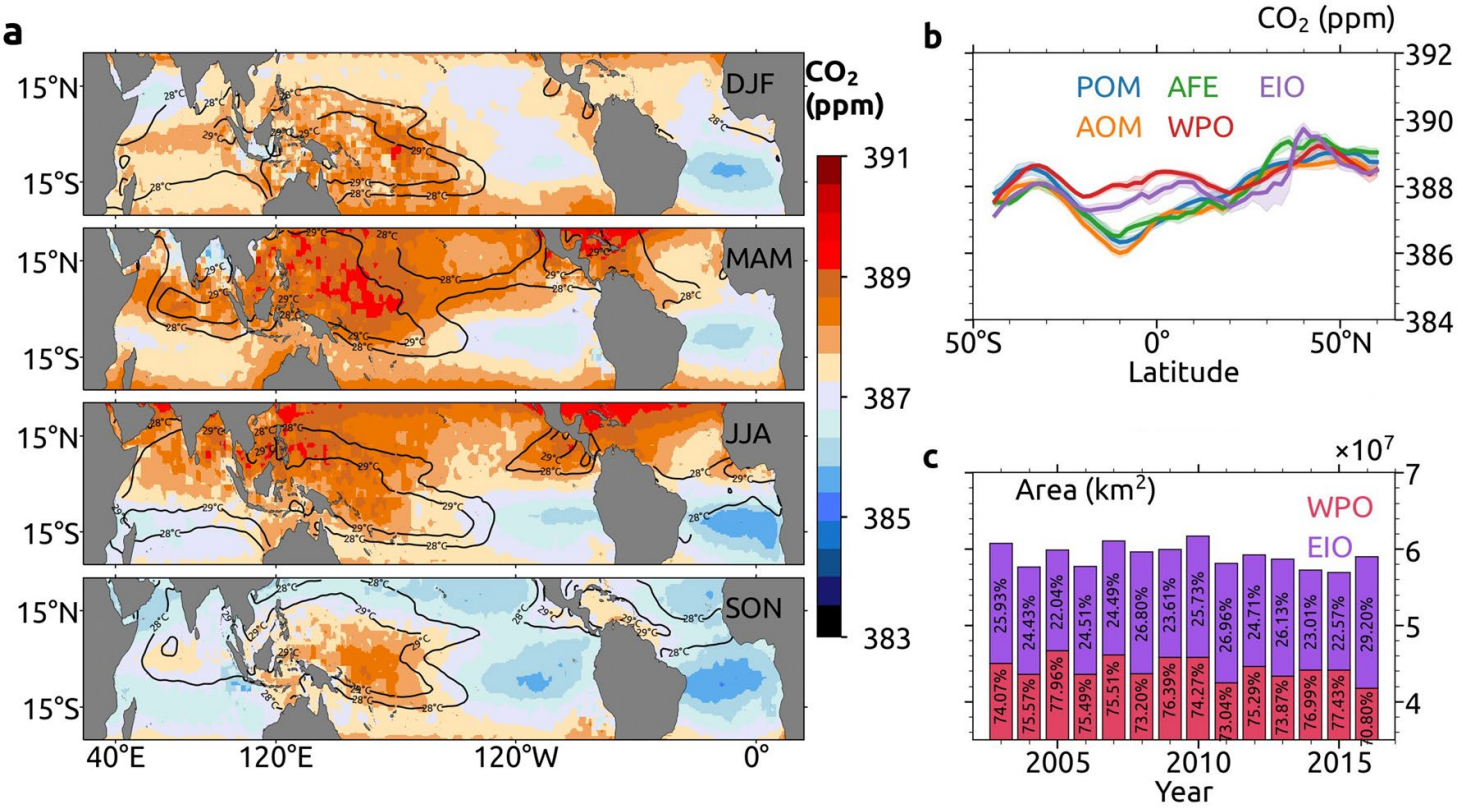
Variability of the CO_2_ pool over the Indo-Pacific Warm Pool (IPWP). (**a**) Seasonal average (DJF: December, January and February, MAM: March, April and May, JJA: June, July and August, SON: September, October and November) mid-tropospheric CO_2_ (ppm) over the tropical oceans for the period 2002–2017. Black contours represent the seasonal average of Sea Surface Temperature (SST) in °C for the period 2002–2017. These maps are generated using Cartopy^64^ 0.18.0 (https://scitools.org.uk/cartopy). (**b**) Latitudinal variability of annual averaged mid-tropospheric CO_2_ over different regions (with 95% confidence interval) such as the Pacific Ocean Minimum (POM), Atlantic Ocean Minimum (AOM), AFE (Africa and Europe, averaged between longitudes 10° E and 45° E), CO_2_ pool over Western Pacific Ocean (WPO) and CO_2_ pool over East Indian Ocean (EIO). (**c**) Yearly variations of the area of CO_2_ pool and contributions of WPO and EIO are indicated. Note that the y-axis starts from 3.5 × 10^7^ km^2^.

Figure 3a shows the seasonal variability of CO_2_ over the tropics, overlaid with SST, which indicates the temporal changes in IPWP. Regions of high CO_2_ concentrations align with the 28–29 °C SST contours in most months. The zonal and meridional extensions of IPWP are also mimicked by the CO_2_ distributions. For example, IPWP extends more to the southern hemisphere in winter, which is also followed by CO_2_. Similarly, the zonal growth of IPWP is partly responsible for the high concentration of CO_2_ that extends to the eastern Pacific in summer, and even to the tropical Atlantic Ocean.

The seasonal variability of CO_2_ is attributed to the changes in its sources, sinks, horizontal winds and vertical transport. The CO_2_ pool over the eastern Indian Ocean shows more visible seasonal changes than that in the western tropical Pacific Ocean. During October–November, the CO_2_ pool is one of the high CO_2_ concentration regions over the oceans, higher than that over the northern hemisphere. Supplementary Figure S1 shows the interannual variability of CO_2_ pool with an average linear trend of 2.17 ppm yr^-1^; indicating a continuous increase of CO_2_ over the region.

The monthly vertical velocity (Pa s^−1^) averaged from 1000 to 300 hPa is shown in Supplementary Figure S2. The upward movement of air favors the transport of CO_2_ to higher altitudes, whereas the sinking of air suppresses CO_2_ to lower altitudes. Latitude-wise distribution of CO_2_ over different regions is shown in Fig. 3b. All regions exhibit two major peaks, whereas West Pacific Ocean (WPO) and EIO have additional peaks in the tropics representing the CO_2_ pool. As the CO_2_ pool lies over the tropics, it extends to both hemispheres with a noticeable seasonal variability. The western Pacific CO_2_ pool spreads over both hemispheres, although the Indian Ocean shows skewness towards the northern hemisphere. To quantify the minimum zones of CO_2_ over the Atlantic and Pacific Oceans, we selected two regions with coordinates 0°–20° S, 50° W–15° E (AOM) and 0°–15° S, 125°–80° W (POM). Figure 3b shows higher CO_2_ in the northern hemisphere, about 3.5 ppm more than in AOM. Similarly, Africa and Europe, averaged between longitudes 10° E and 45° E (AFE) show high CO_2_ in NH high latitudes.

To assess the spatial coverage of CO_2_ pool, we have calculated the year-wise area, as discussed in *Methods* section. Figure 3c shows the yearly total area of CO_2_ pool from 2003 to 2016. The average area of the CO_2_ pool during 2003–2016 is about 5.82 × 10^7^ km^2^, and the largest area is estimated for 2010 (6.04 × 10^7^ km^2^). The yearly percentage contribution of WPO and EIO to CO_2_ pool indicates that WPO is more prominent in size, roughly three-fourths of the total area of CO_2_ pool in all years.

### Influence of climate modes on CO_2_ pool

During the ENSO events, changes in the Walker circulation have a profound influence on the distribution of CO_2_ in the mid-troposphere^37^. Jiang et al.^38^ reported that the mid-tropospheric CO_2_ levels are elevated over the central and repressed over the western Pacific Oceans during the El Niño events. The change in Walker circulation during El Niño favors CO_2_ transport over the central Pacific Ocean, whereas the downwelling air over WPO limits the CO_2_ transport to higher altitudes. We have selected two regions to quantify the differential response of CO_2_ over the eastern and western Pacific Oceans to the ENSO events. The coordinates of these regions are R1: 10° S–10° N, 85°–160° E and R2: 10° S–10° N, 170°–70° W.

**Figure 4:**
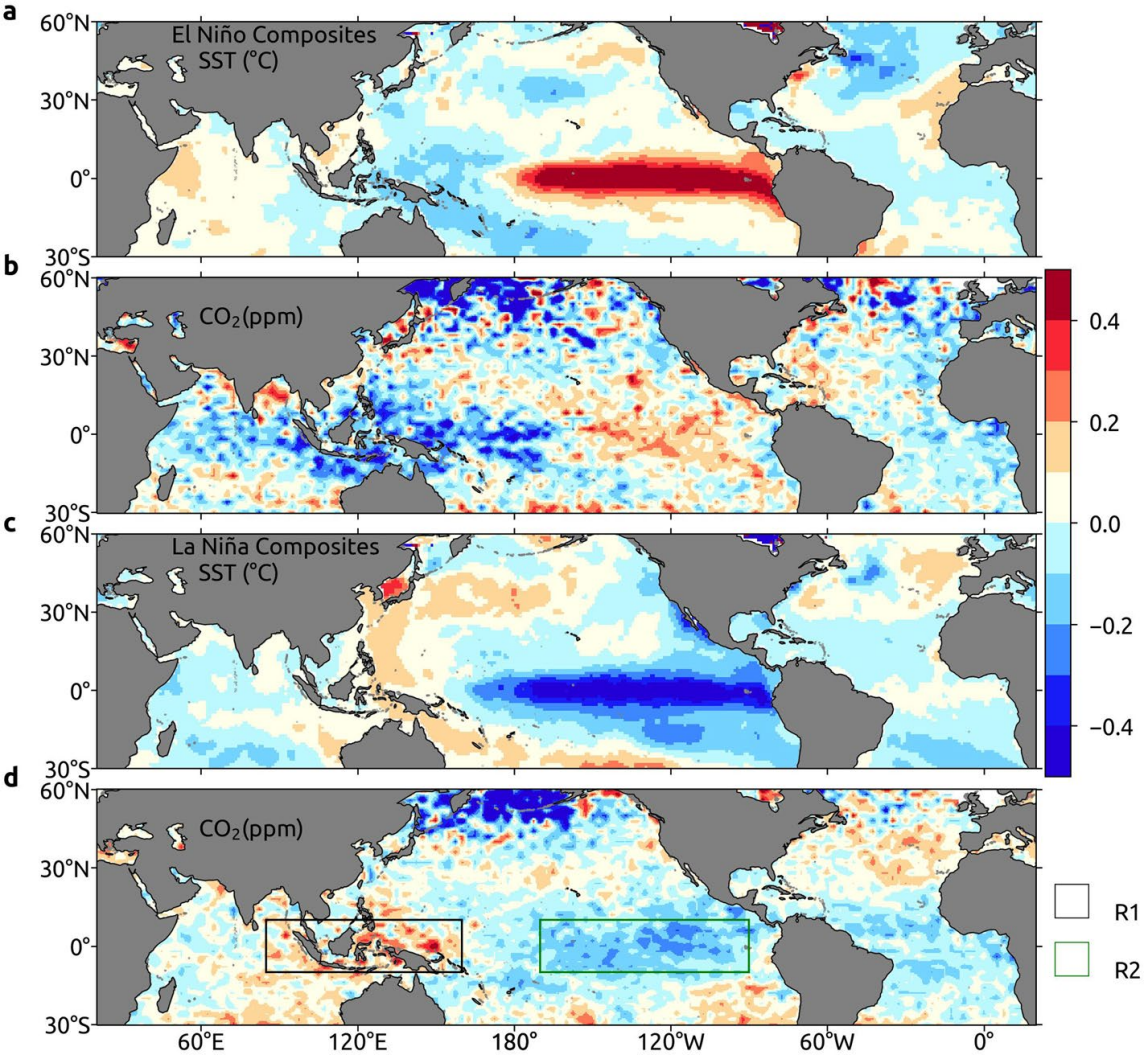
Impact of El Niño and La Niña events. El Niño composites of deseasonalized and detrended (**a**) Sea Surface Temperature (SST) and (**b**) mid-tropospheric CO_2_ during the period 2002–2017. La Niña composites of deseasonalized and detrended (**c**) Sea Surface Temperature (SST) and (**d**) mid-tropospheric CO_2_ during the period 2002–2017. R1 and R2 are the selected regions. These maps are generated using Cartopy^64^ 0.18.0 (https://scitools.org.uk/cartopy).

Liu et al.^39^ showed high CO_2_ emissions during El Niño events due to associated fire activities in south Asia. However, the ENSO composites of the detrended, deseasonalized and latitude-averaged vertical velocity (Supplementary Figure S5) reveal that its negative anomaly (downward motion of air) inhibits the upward transport of high CO_2_ concentration at R1 and thus, make lower CO_2_ in mid-troposphere there. Similarly, positive anomalies of vertical velocities are present over R1 during La Niña events. This upward-moving air transports high CO_2_ to the mid-troposphere from the surface, which gives rise to positive anomaly of CO_2_ over IPWP during strong La Niña events (Fig. 4d). As compared to R1, a contrasting effect is present over R2 owing to the opposite influence of vertical velocity during the La Niña and El Niño events.

The yearly averaged distribution of CO_2_ and SST show high values across the western central Indian Ocean that extends to the southeast Arabian Sea^13^. The region where the warm pool lies is also under the influence of ENSO events (Fig. 4a, c). The response of atmospheric CO_2_ to the ENSO events is identified using the averaged data of respective months (Fig. 4b, d). For instance, the positive SST anomaly over R1 during La Niña corresponds to relatively higher CO_2_ and the negative SST anomaly reciprocates with lower CO_2_ in the mid-troposphere. Similarly, the response of R2 to La Niña and El Niño reiterates the positive relationship between SST and CO_2_ anomalies. Furthermore, the northeast Atlantic Ocean also has a similar response, which replicates R1; indicating the influence of climate modes on SST and CO_2_ in all oceanic regions.

**Figure 5:**
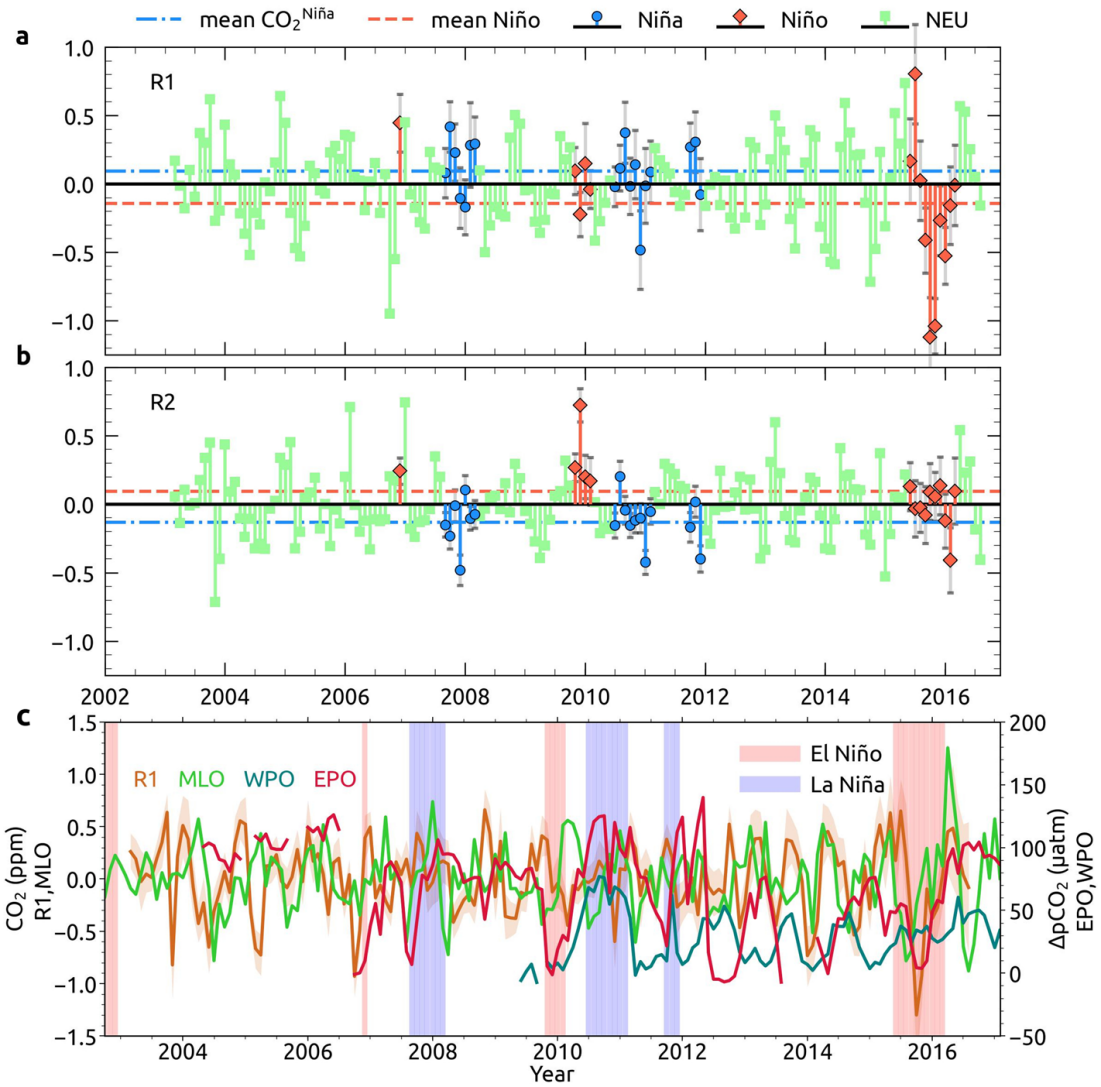
Response of CO_2_ anomalies to ENSO and comparison of AIRS CO_2_ with surface and buoy measurements. (**a**), Detrended and deseasonalized month-wise CO_2_ anomalies (ppm) over the regions R1 and (**b**) for R2 during the period 2002–207. Blue indicates mid-tropospheric CO_2_ during La Niña months (CO_2_^Niña^, ENSO index <  − 1 ), Orange is mid-tropospheric CO_2_ during El Niño months (CO_2_^Niño^, ENSO index >  + 1) and pale green indicates CO_2_ during neutral months and months with ENSO index within ± 1 (CO_2_^neutral^). Regions R1 and R2 are marked in Fig. 4d. Mid-tropospheric CO_2_ during La Niña and El Niño events is shown with 95% confidence intervals and are indicated as error bars (grey). (**c**) Detrended and deseasonalized measurements from Mauna Loa observatory (MLO), ∆pCO_2_ (μatm) from the buoys Chuuk K1, TAO 0°, 165° E, TAO 8° S, 165° E and TAO 0, 170° W representing the western Pacific ocean (WPO) and Stratus 85° W 20° S, TAO 0°, 110° W, TAO 0, 125° W and TAO 0, 140° W represent the eastern Pacific oceanic regions. Detrended and deseasonalized AIRS CO_2_ (at 95% confidence level) over R1 as marked in Fig. 4d is indicated as R1 here. The periods of El Niño and La Niña are shaded.

The interannual variability of CO_2_ anomalies at R1 and R2 is shown in Fig. 5a,b. We have considered only the strong El Niño (December 2006, November 2009–February 2010, June 2015–March 2016) and La Niña (September 2007–March 2008, July 2010–February 2011, October 2011–December 2011) events here. During El Niño and La Niña periods, anomalies of CO_2_ to corresponding SST differences are nearly proportionate, but opposite over the regions R1 and R2. This shows the CO_2_ response to a warming ocean. During the 2015–2016 El Niño, a strong feedback is observed over R1, and CO_2_ anomalies reached − 1.1 ppm. During the neutral and weak ENSO events (ENSO index within ± 1), R1 and R2 show similar CO_2_ peaks; suggesting that those peaks could be the result of the variability in large-scale CO_2_ emissions.

### CO_2_ observations at ocean surface

The near-surface measurements of atmospheric CO_2_ show an immediate and prominent response to changes in carbon sources than CO_2_ in mid-troposphere due to the proximity advantage of surface measurements (Fig. 5c). A study on the influence of Pacific Ocean on atmospheric CO_2_ using measurements from Mauna Loa observatory (MLO) shows that most El Niño events correspond to an immediate decrease in atmospheric CO_2_ within a month or two^40^, which can be also found in **Fig. ****5****c**. The subsequent rise in CO_2_ in the following months can be attributed to the influence of declined CO_2_ intake by the global biosphere, increased plant and soil respiration (0.6 ± 1.01 gigatons C in Africa) and enhanced fire emissions (0.4 ± 0.08 gigatons C in tropical Asia) during the El Niño events^33,39^. Anomalies computed from the satellite observations at R1 and surface measurements at MLO show similar variability in CO_2_ with some lag/lead as illustrated in Fig. 5c. The magnitude of CO_2_ peaks is often very high for MLO and higher for buoy measurements located at the central and eastern tropical Pacific Ocean (EPO) than that of satellite observations. The shallow warm water in IPWP acts as a barrier between the cooler water and atmosphere, and restricts CO_2_ venting there, which is evident from the lower ΔpCO_2_ (difference between surface seawater pCO_2_ and atmospheric pCO_2_) values at WPO than that of EPO (Fig. 5c). There is a reduction in CO_2_ outgassing from EPO and WPO during El Niño events (e.g. ΔpCO_2_ ≈ 0 μatm at EPO), but it enhanced during La Niña events (e.g. ΔpCO_2_ > 70 μatm at EPO and WPO), although not all ENSO events have a notable influence on CO_2_ concentrations in the atmosphere. These differences are probably due to the complex ocean–atmosphere–biosphere coupled interactions^40^. The increased CO_2_ venting during La Niña events contributes to the higher CO_2_ in the mid-troposphere. The outgassed CO_2_ together with other emissions from land regions are transported to higher altitudes by the vertical winds (see Supplementary Figure S4).

### Impact of CO_2_ pool on global climate

**Figure 6:**
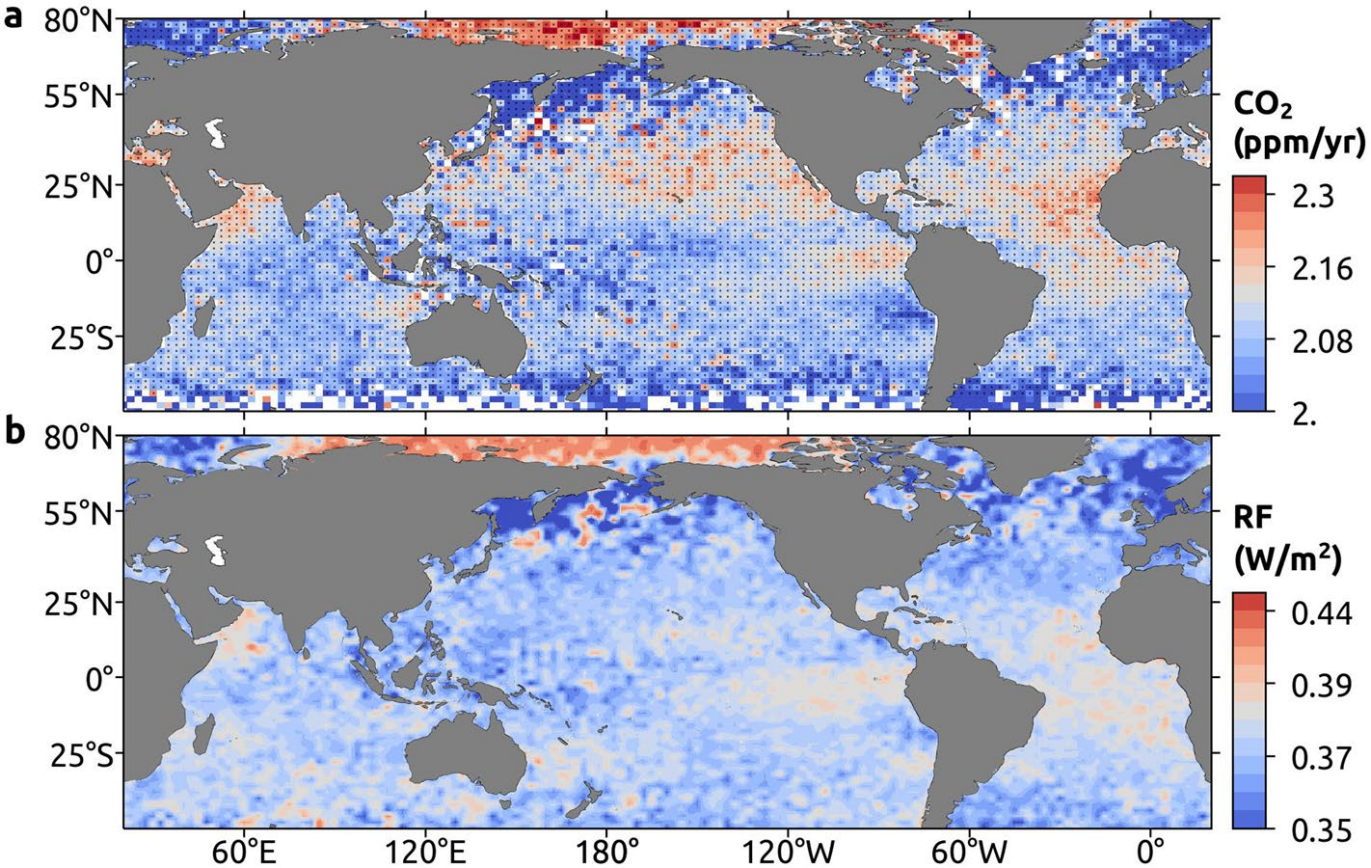
Radiative forcing. (**a**) Annual trend of mid-tropospheric CO_2_ (ppm yr^−1^) during 2003–2016. Stippling indicates significant trends at 95% confidence level. (**b**) Radiative Forcing (RF; W m^−2^) of CO_2_ in 2016. Mid-tropospheric CO_2_ in 2003 is the reference CO_2_ concentration for the calculation of RF here. These maps are generated using Cartopy^64^ 0.18.0 (https://scitools.org.uk/cartopy).

Figure 6a depicts the trend in yearly-averaged mid-tropospheric CO_2_ from 2003 to 2016. Regions north of 60° N show the highest trend values, particularly over the northernmost regions (greater than 2.3 ppm yr^−1^). The eastern Atlantic and Pacific Oceans show higher trends than their regional counterparts. The average annual trend for the CO_2_ pool is 2.17 ppm yr^−1^ (Supplementary Figure S1), which is higher than that of other oceanic regions, e.g. the average trend of CO_2_ over AS is 2.13 ppm yr^−1^ for the period 2003–2016^13^. Nevertheless, the average trend over the region within 20° S–20° N and 50° E–160° W, without the CO_2_ pool criteria, is similar to that of the global average CO_2_ trend (2.11 ppm yr^−1^). The CO_2_ minimum zones over the oceans, AOM and POM, exhibit a high positive trend of CO_2_ (2.14 ± 0.02 ppm yr^−1^ and 2.12 ± 0.02 ppm yr^−1^ respectively). To assess the impact of increased levels of CO_2_ on climate, we also calculate the Radiative Forcing (RF) of CO_2_ in 2016 with respect to that of 2003.

Figure 6b shows the spatial distribution of RF across the regions. High RF is found in regions where CO_2_ concentrations are relatively higher and the average RF at R1 is 0.37 ± 0.01 Wm^−2^. The Atlantic Ocean and WPO exhibit high RF and annual CO_2_ trends (ppm yr^−1^) than those in other oceanic regions. These incessant increase of CO_2_ in the atmosphere exacerbate global warming and this would make it more difficult to keep the warming below 2 °C^2,41^.

## Discussion

A region of high CO_2_ concentration is present over IPWP in the tropics. Temporal (monthly, seasonal and annual) analysis of the CO_2_ data over the region shows that it is a permanent feature. The monthly distribution of SST and mid-tropospheric CO_2_ are congruent, particularly over IPWP. The CO_2_ pool shows an average annual trend of 2.17 ppm yr^−1^, which is comparatively higher than that over other oceanic regions. Several studies have discussed ocean warming in the context of increased GHGs^42,43^. Weller et al.^44^ identified GHG forcing as the major cause of the observed increase in IPWP intensity and size, which produced an increase in ocean heat content and high sea level rise in the twentieth century. Our analysis confirms that the changes in Walker circulation can alter CO_2_ distribution in the mid-troposphere. The transport of surface emissions, both natural and anthropogenic, controlled by atmospheric circulation have distinct seasonal, intra-seasonal and interannual variability. Our assessment suggests that La Niña conditions enhance atmospheric CO_2_ over IPWP, whereas El Niño has a negative effect on it. Apart from these, strong winds in the middle and upper troposphere transport and mix these high concentrations of CO_2_ to the higher altitudes during the periods of ENSO.

Immediate and sharp responses of these climate modes are also well captured by the near-surface and moored buoy CO_2_ measurements. Chatterjee et al.^33^ analyzed the influence of 2015–2016 super El-Niño on atmospheric CO_2_ using the column-averaged measurements from Orbiting Carbon Observatory-2 (OCO-2) and CO_2_ measurements from buoys. They showed an initial drop in atmospheric CO_2_ during the onset of El Niño due to the suppression of upwelling in the tropical Pacific, which reduced the outgassing of CO_2_ from the ocean to atmosphere. The reduction in CO_2_ exchange is accountable for the weak response of CO_2_ anomalies in R2 during the El Niño period, as shown in Fig. 5b.

Furthermore, Indian Ocean Dipole (IOD)^45^ has a comparable effect on mid-tropospheric CO_2_ as that by ENSO^13^. That is, mid-tropospheric CO_2_ exhibits a negative (positive) anomaly during the strong positive (negative) IOD over EIO. During the strong negative IOD in 2016, EIO had the highest contribution to the total area of CO_2_ pool (29.20%) compared to that in other years. A possible reason for this is that the 2015–2016 super El-Niño had already reduced the size of CO_2_ pool over the Pacific Ocean followed by the strongest negative IOD (2016), which favored the uplift of CO_2_ to the mid-troposphere^46,47^. In addition, the combination of strong La Niña and negative IOD favors the upward transport of CO_2_. For instance, a similar event occurred in 2010 and henceforth, the largest CO_2_ pool over IPWP is observed in that year; reiterating the influence of climate modes on the regional and vertical distribution of CO_2_. The atmospheric CO_2_ is gradually increasing, even in the lower CO_2_ regions over the Pacific and Atlantic Oceans and over the CO_2_ pool. The difference between these low CO_2_ regions and the global mean shows a negative trend, which implies that the CO_2_ concentration in the minimum pool is increasing faster than that in other regions. Our analyses also give new insights on the global distribution of CO_2_ in the mid-troposphere and its variability, particularly over the oceanic regions as they are largest carbon sinks on the Earth. Understanding the connection between IPWP and CO_2_ pool is beneficial to tackle the complex ocean–atmosphere interactions and thus, to mitigate global warming and climate change triggered by CO_2_ in the atmosphere.

## Methods

We have used the Atmospheric Infrared Sounder (AIRS) CO_2_ data, which is available from September 2002 to February 2017 with a horizontal resolution of 2.5° × 2°. AIRS is the first hyperspectral Infrared spectrometer on board the Earth Observing System (EOS) Aqua, operating at wavelengths ranging from 3.7 to 15.4 μm^48,49^. Vanish Partial Derivative Method is used to retrieve the mid-tropospheric CO_2_ utilizing a set of 15 μm spectral channels that have peak sensitivities to CO_2_ between 500 and 300 hPa^15,50^. Vanishing Partial Derivatives method utilizes the general property of multivariate total differentials to isolate the contribution of individual minor gases. It minimizes the difference between the observed cloud-cleared and calculated radiances with multiple iterations until the radiance residuals of geographical variables are minimized. Since these satellite data are available only up to 2017, our analysis is performed for the period 2002–2017.

Monthly SST data are obtained from the Hadley Centre Sea Ice and SST dataset (HadISST)^51^, with a 1° × 1° horizontal resolution. HadISST is available from 1871 to date, but we have opted for the same period as that of AIRS CO_2_. Based on Seabold and Perktold^52^, CO_2_ and SST data are detrended and deseasonalized to remove trend and seasonality. The average of satellite measurements over different regions are expressed as the mean ± standard deviation. We use the Ocean Niño Index (ONI) to assess the variability in CO_2_ during ENSO. Composites of CO_2_ and SST are computed for different phases of ENSO. Meyers and O'Brien^40^ indicated that all the ENSO events do not lead to change in CO_2_. Henceforth, we have considered only strong El Niño and La Niña events with the criterion of the 3-month season, with ONI values greater than 1 for El Niño and less than -1 for La Niña months. Among the selected El Niño events, 2015 is the equatorial Pacific and rest are central Pacific El Niño events.

The vertical velocity at pressure levels from 1000 to 300 hPa with a horizontal resolution of 25 × 25 km are taken from the ECMWF Reanalysis version 5 (ERA5) data^53^. The monthly vertical velocity, ω (Pa s^−1^), is scaled by -1 so that positive values indicate upward motion of air masses and negative values indicate downward motion. Longitudes of the regions selected for the meridional average, other than AOM and POM, are 70°–120° E for EIO, 120° E–160° W for WPO and 10°–45° E for representing land region (Africa and Europe). For this analysis, a standard latitude range of 45° S–60° N is selected. For the calculation of the area of CO_2_ pool, we have selected the grid points enclosed over the region within 20° S–20° N and 50° E–160° W, where the anomaly of CO_2_ concentration is greater than its standard deviation but less than three times its standard deviation.

$$CO_{2} \, pool = \sigma < CO_{2}^{20^\circ S - 20^\circ N,\; 50^\circ E - 160^\circ W} < 3\sigma$$

where, σ is the standard deviation of CO_2_ over the region 20° S to 20° N and 50° E to 160° W.

We have also considered the atmospheric and surface seawater partial pressure of CO_2_ (pCO_2_) measured by the open ocean moored buoys. ΔpCO_2_ is calculated by subtracting atmospheric pCO_2_ from surface seawater pCO_2_. We have combined ΔpCO_2_ from Tropical Atmosphere Ocean (TAO) mooring at 0°, 165° E^54^, TAO at 8° S, 165° E^55^, Chuuk K1 mooring at 7.46° N, 151.90° E^56^ and TAO at 0°, 170° W^57^ to represent the western Pacific, whereas Stratus at 85° W, 20° S^58^, TAO at 0°, 110° W^59^ and TAO at 0°, 125° W^60^, TAO at 0°, 140° W^61^ to represent the eastern Pacific based on the mooring locations. Monthly averaged CO_2_ data from the Mauna Loa is also used to supplement the satellite data^62^. It is detrended and deseasonalized based on the method described by Seabold and Perktold^52^.

Since the CO_2_ pool is an area of high concentrations (e.g. 14 years annual average > 385 ppm in the region), we examine the contribution of CO_2_ to regional warming there. Therefore, we have estimated the RF of CO_2_ using the formula^63^,

$$\Delta {\text{F}} = \left( {{5}.{\text{35 W m}}^{{ - {2}}} } \right){\text{ ln}}\left( {{\text{C}}/{\text{C}}_{{\text{O}}} } \right)$$

where, ∆F is the RF for CO_2_ in W m^−2^, C is the CO_2_ concentration based on which RF is calculated. C_O_ is the reference CO_2_ concentration. Conventionally, the pre-industrial level of CO_2_ (280 ppm) is taken as C_O_. However, we consider CO_2_ levels in 2003 as C_O_ to look at the changes in the recent decade. We present the grid-wise radiative forcing and linear trend of CO_2_ to assess the impact of increased CO_2_ levels.

The statistical significance of trend analysis and ENSO composites of vertical velocity is examined using the two-sided student’s *t* test. The difference between El Niño and La Niña composites is calculated and tested for its statistical significance. Grid points with p-values less than 0.05 is considered statistically significant at a 95% confidence level and marked in Supplementary Figure S4. For the ENSO composite analysis of mid-tropospheric CO_2_ and SST, the statistical significance is tested at 90% significant level and the statistically significant points are marked in Supplementary Figure S5. The uncertainties indicated in Fig. 3b, 5 and Supplementary Figure S1 are at 95% confidence intervals.

## Supplementary Information

Supplementary Information.

## Data Availability

AIRS CO_2_ data is publicly available and can be downloaded from https://airs.jpl.nasa.gov. The ERA5 data is acquired from the Copernicus Climate Change Service Information 2020 (https://climate.copernicus.eu/climate-reanalysis. HadISST data are available at the website of Met Office Hadley Centre (https://www.metoffice.gov.uk/hadobs/hadisst/). Moored buoy CO_2_ observations are available at https://oceanacidification.noaa.gov/. Mauna Loa measurements are obtained from https://gml.noaa.gov/ccgg/trends/.

## Code availability

The analysis codes are available on request.

## References

[1] Xu Y, Ramanathan V, Victor DG (2018). Global warming will happen faster than we think. Nature.

[2] IPCC, 2018: Masson-Delmotte, V. et al. Global Warming of 1.5 °C. An IPCC Special Report on the impacts of global warming of 1.5 °C above pre-industrial levels and related global greenhouse gas emission pathways, in the context of strengthening the global response to the threat of climate change, sustainable development, and efforts to eradicate poverty (2018).

[3] Dosio A, Mentaschi L, Fischer EM, Wyser K (2018). Extreme heat waves under 15 °C and 2 °C global warming. Environ. Res. Lett..

[4] Saranya JS, Roxy MK, Dasgupta P, Anand A (2022). Genesis and trends in marine heatwaves over the tropical Indian Ocean and their interaction with the Indian summer monsoon. J. Geophys. Res. Oceans.

[5] Alfieri L (2017). Global projections of river flood risk in a warmer world. Earth's Future.

[6] He Y (2022). Quantification of impacts between 1.5 and 4 °C of global warming on flooding risks in six countries. Clim. Change.

[7] Emanuel K (2005). Increasing destructiveness of tropical cyclones over the past 30 years. Nature.

[8] Kuttippurath J (2022). Tropical cyclone–induced cold wakes in the northeast Indian Ocean. Environ. Sci. Atmos..

[9] Giorgi F (2016). Enhanced summer convective rainfall at Alpine high elevations in response to climate warming. Nat. Geosci..

[10] Kuttippurath J (2021). Observed rainfall changes in the past century (1901–2019) over the wettest place on Earth. Environ. Res. Lett..

[11] Seneviratne, S. et al. *Managing the Risks of Extreme Events and Disasters to Advance Climate Change Adaptation, A Special Report of Working Groups I and II of the Intergovernmental Panel on Climate Change (IPCC)* (eds Field, C.). pp 109–230 (Cambridge University Press, Cambridge, 2012).

[12] Cao L (2019). The global spatiotemporal distribution of the mid-tropospheric CO_2_ concentration and analysis of the controlling factors. Remote Sens..

[13] Peter R, Kuttippurath J, Chakraborty K, Sunanda N (2021). Temporal evolution of mid-tropospheric CO_2_ over the Indian Ocean. Atmos. Environ..

[14] Kuttippurath J, Peter R, Singh A, Raj S (2022). The increasing atmospheric CO_2_ over India: Comparison to global trends. Iscience.

[15] Chahine, M. T. et al. Satellite remote sounding of mid‐tropospheric CO_2_. *Geophys. Res. Lett*. 35 (2008) 10.1029/2008GL035022.

[16] Nemry B, François L, Warnant P, Robinet F, Gérard JC (1996). The seasonality of the CO_2_ exchange between the atmosphere and the land biosphere: a study with a global mechanistic vegetation model. J. Geophys. Res. Atmos..

[17] Dettinger MD, Ghil M (1998). Seasonal and interannual variations of atmospheric CO_2_ and climate. Tellus B..

[18] Houghton E (1996). Climate change 1995: The Science of Climate Change: Contribution of Working Group I to the Second Assessment Report of the Intergovernmental Panel on Climate Change.

[19] Joos F, Plattner GK, Stocker TF, Marchal O, Schmittner A (1999). Global warming and marine carbon cycle feedbacks on future atmospheric CO_2_. Science.

[20] Wyrtki, K. Some thoughts about the west Pacific warm pool. In *Proc. of the western pacific international meeting and workshop on TOGA COARE*. pp. 99–109 (ORSTOM/Nouméa, New Caledonia, 1989).

[21] Graham NE, Barnett TP (1987). Sea surface temperature, surface wind divergence, and convection over tropical oceans. Science.

[22] Kim ST, Yu JY, Lu MM (2012). The distinct behaviors of Pacific and Indian Ocean warm pool properties on seasonal and interannual time scales. J. Geophys. Res. Atmos..

[23] Williams AP, Funk C (2011). A westward extension of the warm pool leads to a westward extension of the Walker circulation, drying eastern Africa. Clim. Dyn..

[24] Kim HR, Ha KJ, Moon S, Oh H, Sharma S (2020). Impact of the Indo-Pacific warm pool on the Hadley, walker, and monsoon circulations. Atmosphere.

[25] McPhaden MJ (2004). Evolution of the 2002/03 El Niño. Bull. Am. Meteorol. Soc..

[26] Ho CR, Yan XH, Zheng Q (1995). Satellite observations of upper-layer variabilities in the western Pacific warm pool. Bull. Am. Meteorol. Soc..

[27] Yan XH, Ho CR, Zheng Q, Klemas V (1992). Temperature and size variabilities of the Western Pacific Warm Pool. Science.

[28] Fasullo J, Webster PJ (1999). Warm pool SST variability in relation to the surface energy balance. J. Clim..

[29] Roxy MK, Ritika K, Terray P, Masson S (2014). The curious case of Indian Ocean warming. J. Clim..

[30] Sunanda N, Kuttippurath J, Peter R, Chakraborty K, Chakraborty A (2021). Long-term trends and impact of SARS-CoV-2 COVID-19 lockdown on the primary productivity of the North Indian ocean. Front. Mar. Sci..

[31] Murray JW, Barber RT, Roman MR, Bacon MP, Feely RA (1994). Physical and biological controls on carbon cycling in the equatorial Pacific. Science.

[32] Francey RJ (1995). Changes in oceanic and terrestrial carbon uptake since 1982. Nature.

[33] Chatterjee A (2017). Influence of El Niño on atmospheric CO_2_ over the tropical Pacific Ocean: Findings from NASA’s OCO-2 mission. Science.

[34] Olivier, J. G. J., Janssens-Maenhout, G., Peters, J. A. H. W. & Wilson, J. Long-Term Trend in Global CO_2_ Emissions. (Report for PBL Netherlands Environmental Assessment Agency, The Hague, 2011)

[35] Gatti LV (2021). Amazonia as a carbon source linked to deforestation and climate change. Nature.

[36] Jiang X (2013). Influence of El Niño on mid-tropospheric CO_2_ from Atmospheric Infrared Sounder and model. J. Atmos. Sci..

[37] Julian PR, Chervin RM (1978). A study of the Southern oscillation and walker circulation phenomenon. Mon. Weather Rev..

[38] Jiang X, Chahine MT, Olsen ET, Chen LL, Yung YL (2010). Interannual variability of mid-tropospheric CO_2_ from Atmospheric Infrared Sounder. Geophys. Res. Lett..

[39] Liu J (2017). Contrasting carbon cycle responses of the tropical continents to the 2015–2016 El Niño. Science.

[40] Meyers SD, O'Brien JJ (1995). Pacific ocean influences atmospheric carbon dioxide. Eos.

[41] Peters GP (2013). The challenge to keep global warming below 2 C. Nat. Clim. Change.

[42] Nagelkerken I, Connell SD (2015). Global alteration of ocean ecosystem functioning due to increasing human CO_2_ emissions. Proc. Natl. Acad. Sci. U. S. A..

[43] Heede UK, Fedorov AV (2021). Eastern equatorial Pacific warming delayed by aerosols and thermostat response to CO_2_ increase. Nat. Clim. Change.

[44] Weller E (2016). Human-caused Indo-Pacific warm pool expansion. Sci. Ad..

[45] Saji NH, Goswami BN, Vinayachandran PN, Yamagata T (1999). A dipole mode in the tropical Indian Ocean. Nature.

[46] Chen L, Li T, Wang B, Wang L (2017). Formation mechanism for 2015/16 super El Niño. Sci. Rep..

[47] Lu B (2018). An extreme negative Indian ocean dipole event in 2016: Dynamics and predictability. Clim. Dyn..

[48] Aumann H (2003). AIRS/AMSU/HSB on the aqua mission: Design, science objectives, data products, and processing systems. IEEE Trans. Geosci. Remote Sens..

[49] Pagano TS, Aumann HH, Hagan DE, Overoye K (2003). Prelaunch and in-flight radiometric calibration of the atmospheric infrared sounder (AIRS). IEEE Trans. Geosci. Remote Sens..

[50] Chahine, M., Barnet, C., Olsen, E. T., Chen, L. & Maddy, E. On the determination of atmospheric minor gases by the method of vanishing partial derivatives with application to CO_2_. *Geophys. Res. Lett*. 32 (2005).

[51] Rayner N (2003). Global analyses of sea surface temperature, sea ice, and night marine air temperature since the late nineteenth century. J. Geophys. Res. Atmos..

[52] Seabold, S. & Perktold, J. S. econometric and statistical modeling with Python. In *Proc. of the 9th Python in Science Conf.* 57–61 (2010).

[53] Hersbach H (2020). The ERA5 global reanalysis. Q. J. R. Meteorol. Soc..

[54] Sutton, Adrienne J. et al. High-resolution ocean and atmosphere pCO_2_ time-series measurements from mooring TAO165E0N in the Equatorial Pacific Ocean (NCEI Accession 0113238). ). *NOAA Natl. Cent. Environ. Inf.* https://doi.org/10.3334/cdiac/otg.tsm_tao165e0n (2013a).

[55] Sutton, Adrienne J. et al. High-resolution ocean and atmosphere pCO_2_ time-series measurements from mooring TAO165E8S in the Equatorial Pacific Ocean (NCEI Accession 0117073). *NOAA Natl. Cent. Environ. Inf.* https://doi.org/10.3334/cdiac/otg.tsm_tao165e8s (2014).

[56] Sutton, Adrienne J. et al. High-resolution ocean and atmosphere pCO_2_ time-series measurements from mooring ChuukK1_152E_7N in the North Pacific Ocean (NCEI Accession 0157443). *NOAA Natl. Cent. Environ. Inf.* https://doi.org/10.3334/cdiac/otg.tsm_chuukk1_152e_7n (2016).

[57] Sutton, Adrienne J. et al. High-resolution ocean pCO_2_ time-series measurements from mooring TAO170W_0N in the Equatorial Pacific Ocean (NCEI Accession 0100078). *NOAA Natl. Cent. Environ. Inf.* https://doi.org/10.3334/cdiac/otg.tsm_tao170w_0n (2012b).

[58] Sutton, Adrienne J. et al. High-resolution ocean and atmosphere pCO_2_ time-series measurements from mooring Stratus_85W_20S in the South Pacific Ocean (NCEI Accession 0100075). *NOAA Natl. Cent. Environ. Inf.* https://doi.org/10.3334/cdiac/otg.tsm_stratus_85w_20s (2012a).

[59] Sutton, Adrienne J. et al. High-resolution ocean and atmosphere pCO_2_ time-series measurements from mooring TAO110W_0N in the Equatorial Pacific Ocean (NCEI Accession 0112885). *NOAA Natl. Cent. Environ. Inf.* https://doi.org/10.3334/cdiac/otg.tsm_tao110w (2013b).

[60] Sutton, A. J. et al. High-resolution ocean and atmosphere pCO_2_ time-series measurements from mooring TAO125W_0N in the Equatorial Pacific Ocean (NCEI Accession 0100076). *NOAA Natl. Cent. Environ. Inf*. https://doi.org/10.3334/cdiac/otg.tsm_tao125w (2012c).

[61] Sutton, A. J. et al. High-resolution ocean and atmosphere pCO_2_ time-series measurements from mooring MOORING TAO140W_0N in the Equatorial Pacific Ocean (NCEI Accession 0100077). *NOAA Natl. Cent. Environ. Inf*. https://doi.org/10.3334/cdiac/otg.tsm_tao140w (2012d).

[62] Keeling CD (1976). Atmospheric carbon dioxide variations at Mauna Loa observatory. Hawaii. Tellus.

[63] Myhre G, Highwood EJ, Shine KP, Stordal F (1998). New estimates of radiative forcing due to well mixed greenhouse gases. Geophys. Res. Lett..

[64] Met Office UK (2010). Cartopy: A Cartographic Python Library with a Matplotlib Interface.

