# Supplementary material for: A high concentration CO2 pool over the Indo-Pacific Warm Pool
Source: Sci Rep. 2023 Mar 15;13:4314. doi: 10.1038/s41598-023-31468-0 (PMC10017811; doi:10.1038/s41598-023-31468-0)
Supplement: Supplementary file 1 — Supplementary Information. [file 41598_2023_31468_MOESM1_ESM.pdf]

SUPPLEMENTARY DATA

A high concentration CO_2 pool over the Indo-Pacific Warm Pool

R. Peter[1], J. Kuttippurath[1*], Kunal Chakraborty[2], N. Sunanda[1]

[1]CORAL, Indian Institute of Technology Kharagpur, Kharagpur–721302, India

[2]Indian National Centre for Ocean Information Services, Ministry of Earth Sciences, Hyderabad, India

* To whom correspondence should be addressed. Email: jayan@coral.iitkgp.ac.in

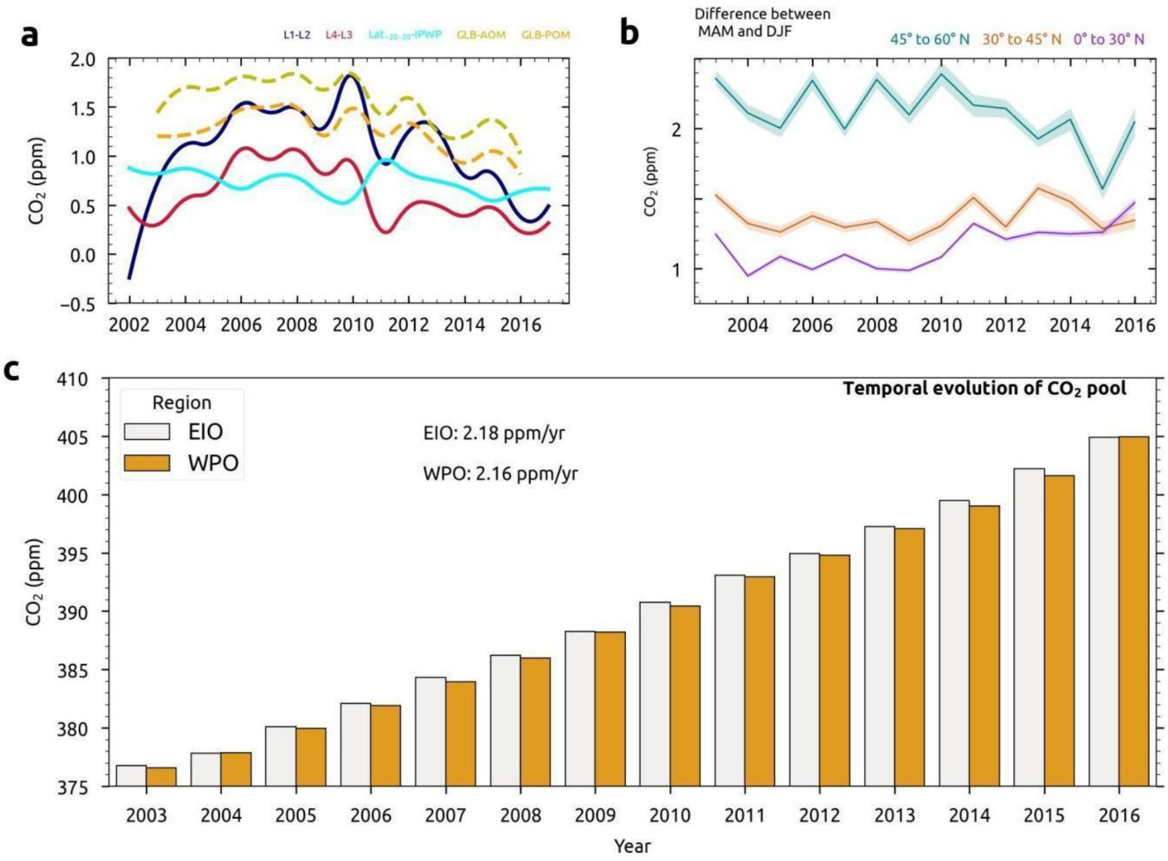

Figure S1: Interannual variations of CO_2 pool. Temporal evolution of (**a**) difference between mean mid-tropospheric CO_2 over different regions (L1: 30–60° N, L2: 0°–30° N, L3: 30° S–0° and L4: 45° S–30° S, $Lat_{-20\text{-}20}$-IPWP is the difference between mid-tropospheric CO_2 over 20° S-20° N and Indo-Pacific Warm Pool, GLB: Global, AOM: Atlantic Ocean Minimum, POM: Pacific Ocean Minimum). (**b**) seasonal difference (March, April and May minus December, January and February) of mid-tropospheric CO_2 over different latitude regions (45° to 60° N, 30° to 45° N and 0° to 30° N). Shading indicates 95 % confidence intervals. (**c**) CO_2 pool over regions– East Indian Ocean (EIO) and West Indian Ocean (WIO) from 2003 to 2016. Annual trends are also mentioned.

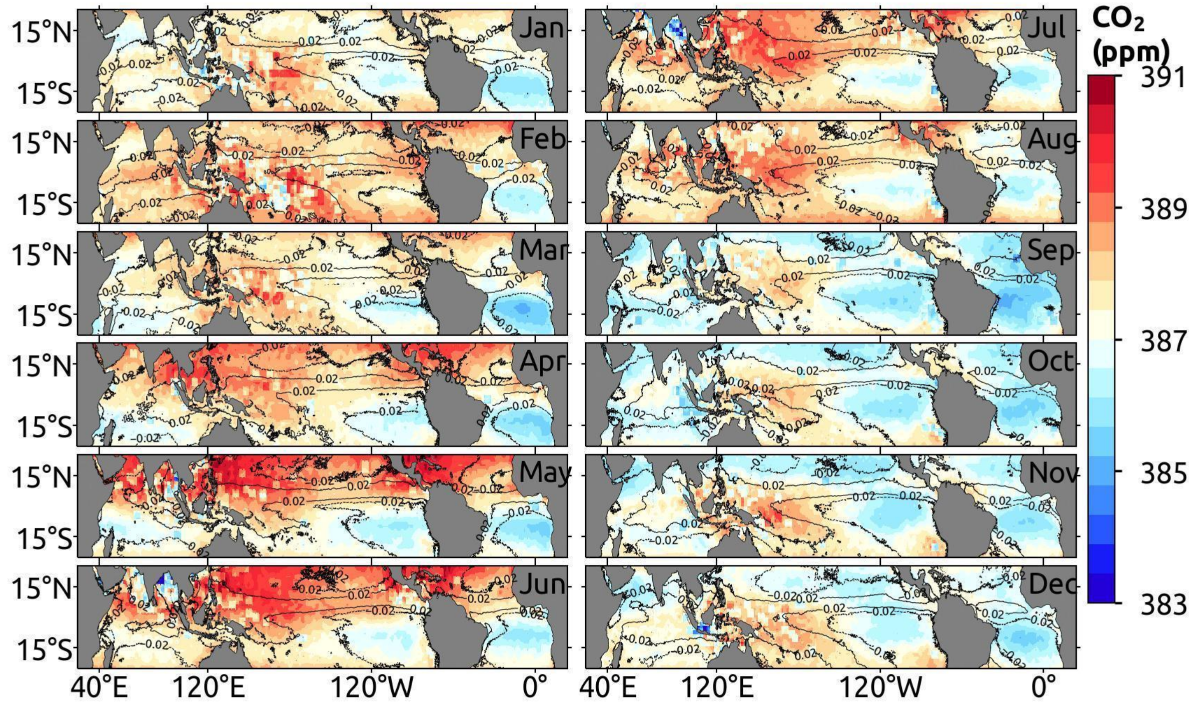

Figure S2: Monthly variations of Carbon dioxide (CO_2) pool over the Indo-Pacific Warm Pool (IPWP). Mid-tropospheric CO_2 (ppm) over the tropical oceans for all months averaged for 2002–2017. Black contours are the monthly mean vertical velocity (Pa s^{-1}) from 1000 hPa to 300 hPa during 2002–2017. Vertical velocity is scaled by -1 for better representation. Thus, here positive values of vertical velocity indicate upward motion and negative values indicate downward motion. Negative contours are marked with dashed lines. The maps were generated using Cartopy[1] (https://scitools.org.uk/cartopy).

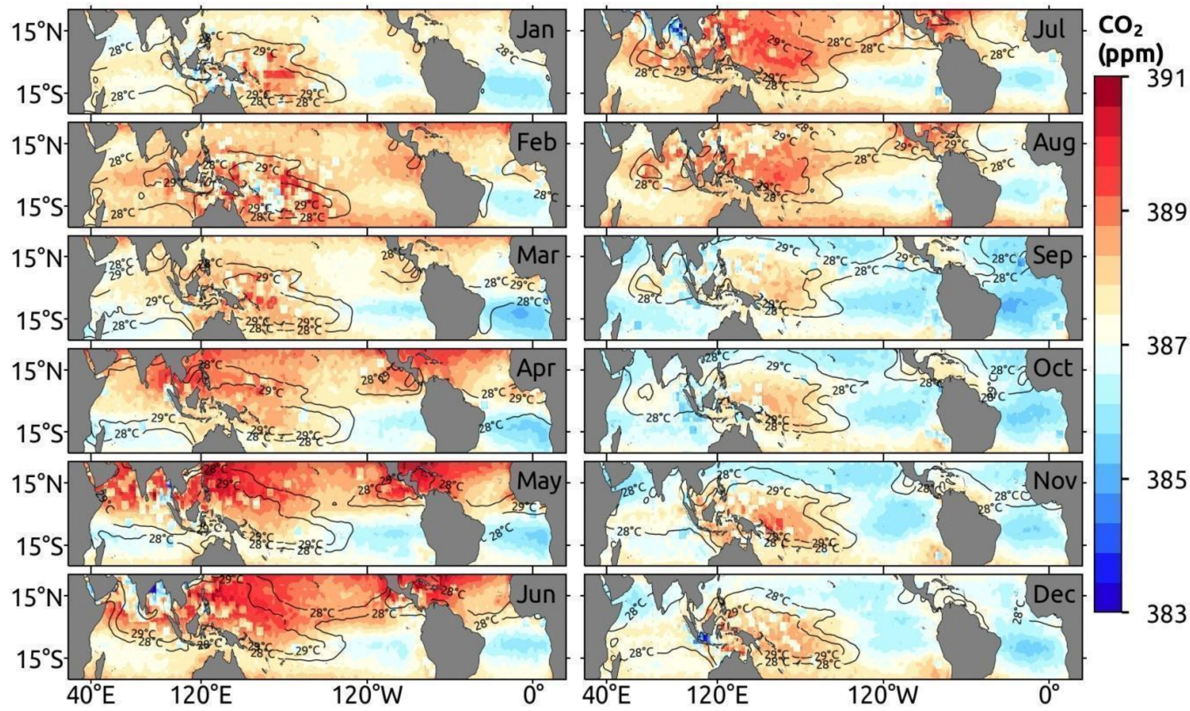

Figure S3: Monthly variations of Carbon dioxide (CO_2) pool over the Indo-Pacific Warm Pool (IPWP). Mid-tropospheric CO_2 (ppm) over the tropical oceans for all months averaged for 2002–2017. Black contours represent the Sea Surface Temperature (SST) in °C during the same period. Land regions are masked. These maps were generated using Cartopy[1] (https://scitools.org.uk/cartopy).

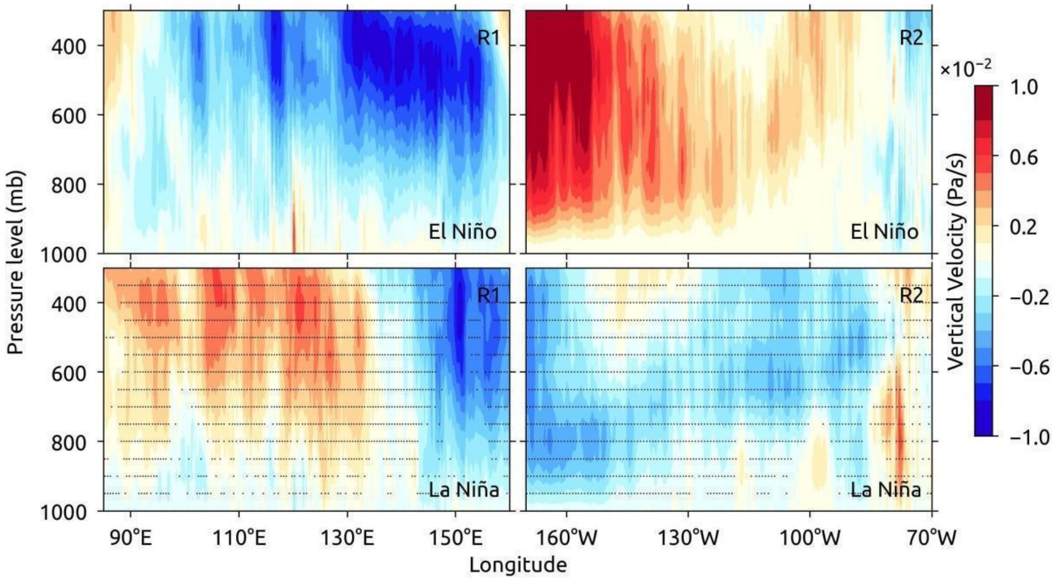

Figure S4: ENSO composites of vertical velocity. El Niño and La Niña composites of deseasonalized and detrended Vertical Velocity (Pa s^{-1}) over R1 and R2. Student's t-test is carried out to test the significance of the difference between the ENSO composites and its significance is marked in the bottom panels for R1 and R2. Stippling indicates significance at 95 % confidence level.

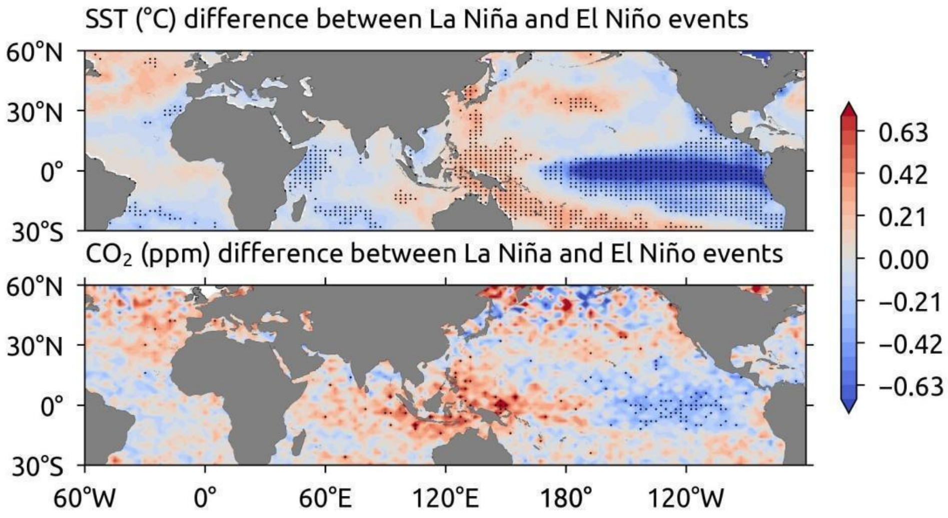

Figure S5: Difference between ENSO composites and its significance. Difference between the El Niño and La Niña composites of Sea Surface Temperature (SST) and mid-tropospheric CO_2 over tropical oceans. Student's t-test is carried out to quantify the statistical significance of the difference and the statistically significant regions at 90 % confidence level are stippled in the figure. These maps were generated using Cartopy[1] (https://scitools.org.uk/cartopy).

References

1. Met Office, U. K. Cartopy: A cartographic python library with a matplotlib interface. Exeter, Devon (2010).
